# Whole-Genome Sequence of *Mycobacterium bovis* W-1171, Isolated from the Laryngopharyngeal Lymph Node of a Wild Boar in South Korea

**DOI:** 10.1128/genomeA.01464-15

**Published:** 2015-12-10

**Authors:** Narae Kim, Yunho Jang, So-young Park, Woong-seog Song, Jong-Taek Kim, Hee Soo Lee, Young-Hee Lim, Jae-Myung Kim

**Affiliations:** a Bacterial Disease Division, Animal and Plant Quarantine Agency, Anyangsi, Gyeonggido, Republic of Korea; b Department of Integrated Biomedical and Life Science, College of Health Science, Korea University, Seoul, Republic of Korea; c Gyeonggi Province Northern Livestock & Veterinary Service, North Branch, Namyangjusi, Gyeonggido, Republic of Korea; d College of Veterinary Medicine, Kangwon National University, Chuncheonsi, Kangwondo, Republic of Korea

## Abstract

*Mycobacterium bovis* W-1171 was isolated from a wild boar living in a free-ranging field in Gyeonggido, South Korea. The whole-genome sequence of this strain was determined in 50 contigs, which was 4,304,865 bp with a 65.57% G+C content. In total 3,945 protein-coding genes were predicted from this assembly.

## GENOME ANNOUNCEMENT

Wildlife, including raccoon dogs, water deer, and wild boar, is an important reservoir of *Mycobacterium bovis*, the causal agent of bovine tuberculosis (bTB). Wild animals are also capable of transmitting the disease to livestock such as cattle (1, 2). To understand the transmission and maintenance of *M. bovis* in various animal species, its pathogenicity mechanism should be determined through comparisons to other sequenced strains (3). Here, we present the whole-genome sequence of *M. bovis* strain W-1171 to provide additional information about *M. bovis* strains isolated from wild animals and to better understand their characteristics. W-1171 was isolated from the laryngopharyngeal lymph node of a bTB-infected wild boar captured near cattle herds in Gyeonggido, South Korea. This strain has the SB1040 spoligotype, which is one of the most commonly found types in South Korea, and has a 53375431052 mycobacterial interspersed repetitive unit-variable-number tandem-repeat pattern.

The genome sequence was obtained using a combination of the 300-bp pair-ended Illumina MiSeq (13,110.87 reads; 670.52× coverage) and Roche 454 GS FLX Titanium systems with an 8-kb paired-end library (310,165.58 reads; 13.83× coverage). Sequencing analysis was performed by Chunlab, Inc. (Seoul, Republic of Korea). The hybrid genome assembly was achieved using GS Assembler 2.6 (Roche), CLC Genomics Workbench 7.0.4 (CLCbio), and CodonCode Aligner (CodonCode Co.). Gene annotation was performed using the NCBI Prokaryotic Genomes Annotation Pipeline (http://www.ncbi.nlm.nih.gov/genome/annotation_prok/) (4).

The final assembly contains 50 contigs (3,945 protein-coding sequences, 4,304,865 bp with a 65.57% G+C ratio), 46 tRNA genes, and 3 rRNA operons. Except for the genes predicted to be related to general and unknown functions, most of the open reading frames (ORFs) matched genes related to “transcription” (233 ORFs, 6.57%) according to eggNOG functional categories (5). Genes related to “lipid transport and metabolism” and “energy production and conversion” ranked the second and third (216 ORFs, 6.09% and 210 ORFs, 5.92%, respectively). Other *M. bovis* strains such as AF2122/97 (GenBank accession number NC_002945), BCG Pasteur 1173P2 (NC_008769), and Mb1595 (CP012095, previously sequenced in our laboratory) (6) showed the same ranking order of ORFs in eggNOG analysis. Mb1595 additionally showed third-ranked ORFs related to “replication, recombination, and repair.” These strains likely showed a high proportion of lipid-related genes because the mycobacterial cell wall lipids are considered to be important factors for their pathogenesis and virulence (7). For example, the transmembrane transport proteins MmpL and MmpS are responsible for transporting cell wall lipids across the mycobacterial membrane, drug resistance, and iron acquisition (7), which are major virulence factors in mycobacteria that are conserved in *M. bovis* and *M. tuberculosis*. In addition, the lipid-rich cell wall enables mycobacteria to survive under adverse conditions such as nutrient shortage and antimicrobial exposure (8, 9). Lastly, clustering analysis based on average nucleotide identity using EzGenome (http://www.ezbiocloud.net/ezgenome/ani) revealed that strain W-1171 was closely related to Mb1595 (99.99%). This genome sequence will serve as a valuable reference for understanding the variation in virulence and epidemiological traits among *M. bovis* strains.

### Nucleotide sequence accession number.

The whole-genome sequence of *M. bovis* strain W-1171 has been deposited at GenBank under the accession number JXTK00000000.
